# Supplementary figures and images for: Untangling the taxonomic knot of Croton anomalus (Euphorbiaceae), a Neotropical dry forest shrub
Source: PeerJ. 2025 Apr 1;13:e19176. doi: 10.7717/peerj.19176 (PMC11970418; doi:10.7717/peerj.19176)

Figure S1  
*trnL-trnF* tree

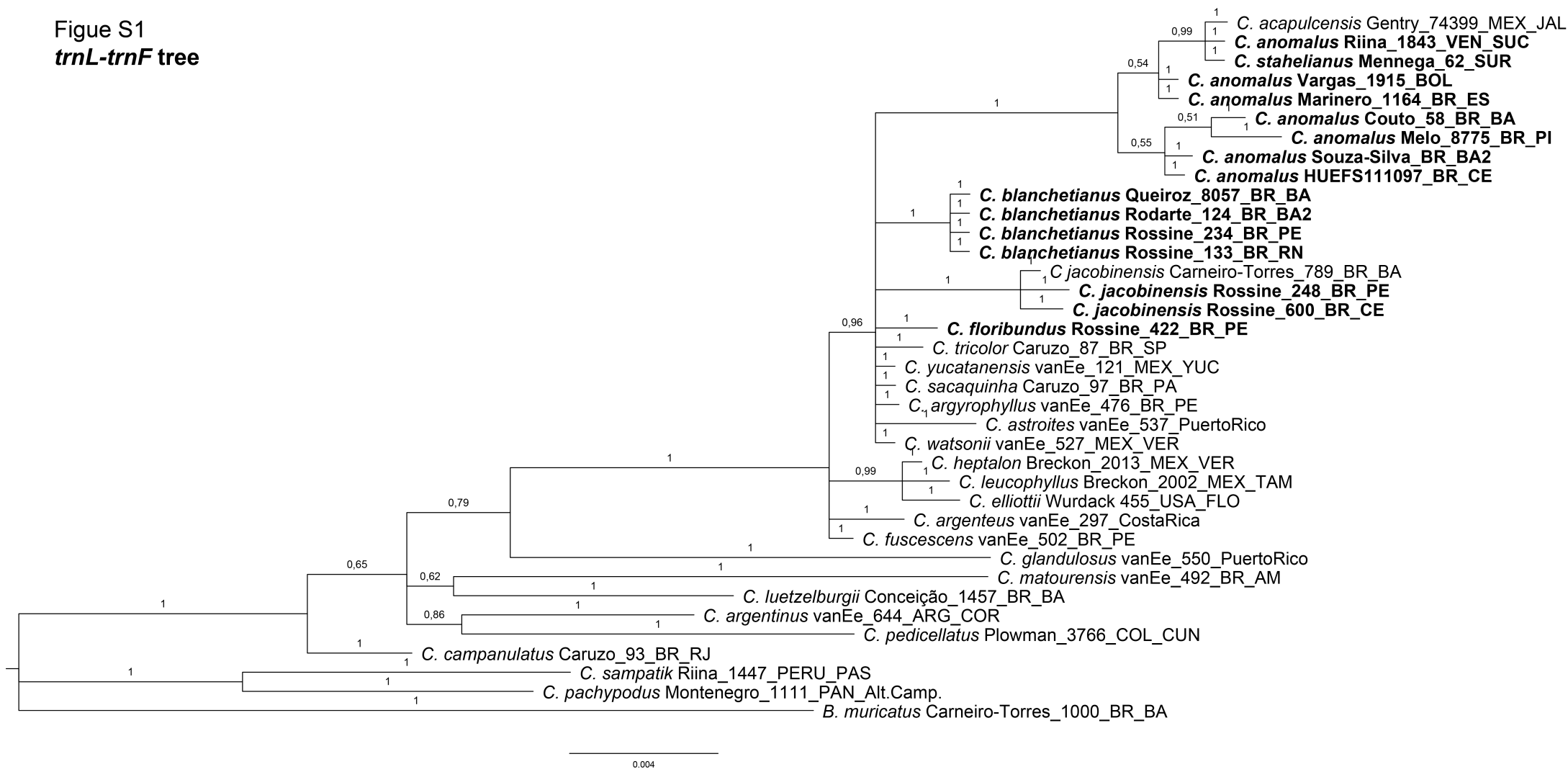

Supplement: Supplemental Information 3 — Phylogenetic reconstruction of the Croton anomalus group illustrated by a majority consensus tree obtained from the Bayesian analysis of the ITS dataset. Names in bold are those newly generated in this study. [file peerj-13-19176-s003.pdf]
